# Brain Activity During Antisaccades to Faces in Adolescence

**DOI:** 10.1093/texcom/tgab057

**Published:** 2021-09-24

**Authors:** Alia Afyouni, Franziska Geringswald, Bruno Nazarian, Marie-Helene Grosbras

**Affiliations:** Aix-Marseille Université, CNRS, LNC, Marseille, France; Aix-Marseille Université, CNRS, LNC, Marseille, France; Aix Marseille Université, CNRS, INT Institut des Neurosciences de la Timone, UMR 7289, Centre IRM-INT@CERIMED, Marseille, France; Aix-Marseille Université, CNRS, LNC, Marseille, France

**Keywords:** adolescence, cognitive control, development, face perception, neuroimaging

## Abstract

Cognitive control and social perception both change during adolescence, but little is known of the interaction of these 2 processes. We aimed to characterize developmental changes in brain activity related to the influence of a social stimulus on cognitive control and more specifically on inhibitory control. Children (age 8–11, *n* = 19), adolescents (age 12–17, *n* = 20), and adults (age 24–40, *n* = 19) performed an antisaccade task with either faces or cars as visual stimuli, during functional magnetic resonance brain imaging. We replicate the finding of the engagement of the core oculomotor and face perception brain regions in all age-groups, with increased involvement of frontoparietal oculomotor regions and fusiform face regions with age. The antisaccade-related activity was modulated by stimulus category significantly only in adolescents. This interaction was observed mainly in occipitotemporal regions as well as in supplementary motor cortex and postcentral gyrus. These results indicate a special treatment of social stimuli during adolescence.

## Introduction

Adolescence is the transitional stage between childhood and adulthood: from puberty to independence from family. It is characterized by changes in cognitive control as well as changes in social behavior and social roles. These are paralleled by neurodevelopmental changes in brain structures (Giedd et al. 1999; Lebel et al. 2008) and functional organization (Menon 2011), with important interregional variations with regards to timing. Notably, brain areas involved in cognitive control and brain areas involved in face and socio-emotional processing follow delayed maturation well into adolescence (review in Blakemore 2008; Crone and Dahl 2012)*.* Here we investigated the functional integration between these 2 networks during adolescent development. We focused on a specific component of cognitive control: inhibitory control.

Many laboratory tasks have been employed to study inhibitory control. The antisaccade, stop-signal, Go/Nogo, flanker, and Stroop tasks all require the capacity to inhibit a prepotent response. In all these tasks, performance improves during childhood and early adolescence (Rubia et al. 2007; rev in Diamond 2013) going along with an increased engagement of prefrontal, frontal, and parietal regions with age (Luna et al. 2001; Rubia et al. 2006; Sweeney et al. 2007; Velanova et al. 2008).

Here, we used the antisaccade task (Hallett 1978), which is particularly well suited to study the interaction between inhibitory control and social perception across a broad range of ages. In the antisaccade task, participants are asked to inhibit their reflexive eye movement to an abruptly appearing peripheral visual target and to reprogram a saccade in the opposite direction. Readily performed by children and adolescents with good measures reliability (Malone and Iacono 2002; Klein and Fischer 2005), this task is well adapted to studying brain–behavior relationships in these populations (for review see Luna et al. 2008). Furthermore, making eye movements toward a stimulus does not involve an additional modality requiring mapping of the visual stimulus to a discrete response, such as pressing a button, and thus minimizes the influence of unwanted motion in the environment of a magnetic resonance imaging (MRI) scanner. Developmental studies using the antisaccade paradigm have identified several parameters to characterize the maturation of inhibitory control, notably a speeded reaction time as well as time to correct inhibitory errors with age and, importantly, a reduction of inhibitory errors (antisaccade direction errors) with age with a stabilization of the effect around 14–15 years of age (Munoz et al. 1998; Luna et al. 2001; Velanova et al. 2008; rev in Karatekin 2007). Developmental functional magnetic resonance imaging (fMRI) studies are consistent in showing that the basic elements of oculomotor and attention circuitry such as the frontal eye fields (FEFs), supplementary eye fields (SEFs), intraparietal sulcus (IPS), lateral occipital cortex (LOC), and insula present increased activity during antisaccades compared to prosaccades in children like in adults (Luna et al. 2001; Sweeney et al. 2007; Constantinidis and Luna 2019). Data indicate that the extent and strength of activation in most of these regions increase with age during late childhood and adolescence (Luna et al. 2001; Paulsen et al. 2015; Quach et al. 2020; but see Padmanabhan et al. 2011; Ordaz et al. 2013). In addition, the recruitment of the dorsal anterior cingulate cortex (dACC), known to be involved in error monitoring, increases between late childhood and adulthood, indicating the maturation of inhibitory control (Velanova et al. 2008).

In this study, we were interested in the interaction between social perception and inhibitory control across development, and more precisely we were interested in how social cues modulate inhibitory control when inhibitory control is still not fully matured. This question can be addressed with a version of the antisaccade task that uses different categories of stimuli as targets. Using faces, cars, or a noise pattern stimulus, Morand et al. (2010) showed that participants had more difficulty inhibiting their reflexive saccade when the stimulus presented was a face, demonstrating that faces have a prevalent effect over other stimuli on cognitive control. Using a similar paradigm but presenting only faces and cars in an fMRI study, Salvia et al. (2020) found that performing the antisaccade task with a social context goes along with a modulation of brain activity in areas known to be involved in selective reorienting in adults, as indicated by an increased antisaccade-related activity in the superior frontal sulcus for faces. In addition, face-sensitive activity in posterior regions was also modulated as a function of whether participants performed an antisaccade or a prosaccade.

Social perception continues to mature during adolescence. In particular, face processing continues to improve until late adolescence (Scherf and Scott 2012) and face selective brain areas, notably in the fusiform face area (FFA), continue to increase in size and specificity (Scherf et al. 2007, 2014; Peelen et al. 2009). In a behavioral study using the same stimuli as in Morand et al. (2010), we investigated whether this maturation is accompanied by changes in the control of spatial orienting in social contexts during late childhood and adolescence (Geringswald et al. 2020). A core finding of the study was that the previously reported face-effect in the antisaccade task could only be observed starting in midadolescence, and that younger participants made the same percentage or errors for faces and other visual objects (like cars).

Here we intended to extend these results by investigating brain activity during antisaccades to faces or cars in children, adolescents, and adults in regions involved in antisaccades (inhibitory control) and regions involved in face processing (social perception). We aimed to understand the influence of social stimuli on inhibitory control during development, but put in context of both the development inhibitory control and social perception. We therefore tested whether we could: 1) replicate previous developmental brain imaging results concerning the antisaccade network using social stimuli as targets rather than common meaningless stimuli; 2) replicate previous developmental brain imaging findings concerning the face network using an orienting task and peripheral stimuli, rather than common passive observation of central stimuli; and 3) critically, whether a modulation of activity during antisaccades as a function of the social nature of the stimulus could be observed in these brain areas, in different age-groups. To this end, we recruited participants pertaining to 3 age-groups: children (8–11), adolescents (12–17), and adults (24–40). They performed an antisaccade fMRI experiment during which they viewed 2 types of stimuli: faces (social stimuli) or cars (nonsocial stimuli).

Based on previous findings on the maturation of brain areas involved in inhibitory control or face processing, we expected to find more engagement in adults than adolescents and more engagement in adolescents than children in the FEF, superior parietal lobule (SPL), and dACC (as in Luna et al. 2010 and Alahyane et al. 2014) for the task effect (antisaccades/prosaccades), as well as in the face network for the effect of stimulus (faces/cars), notably in the FFA. Furthermore, we expected that the stimulus category would modulate antisaccade-related activity only in adolescents and adults given our previous behavioral results (Geringswald et al. 2020), and potentially in different regions, reflecting differences in how these 2 age-groups process social information. We expected to observe not only differences in activity, but also differences in patterns of activity reflecting local “neuronal representation” of stimuli. To explore this, we extended the analysis with a multivoxel pattern analysis (MVPA) decoding approach, probing for changes in regional patterns of activity as a function of task and their modulation by stimulus category. We expected a more stable representation (i.e., higher decoding from brain activity patterns) of task and stimulus with age. In addition, we expected the task representation to be modulated by the stimulus category only in adolescents and adults.

## Materials and Methods

### Subjects

We recruited 39 children and adolescents through local advertising and word-of-mouth. All came from middle- to high-income families. We also recruited 19 adults through adverts posted on the university campus. To be recruited, participants or their legal guardians had to certify that they had no contraindication to MRI (absence of metallic implants or MRI-incompatible prosthesis, no claustrophobia), they had no history of neurological or mental disorder, and they also had to have normal or corrected-to-normal vision and no signs of color blindness. They described themselves as right-handed, except for 2 left-handed adults. Participants as well as the legal guardians of minors received information explaining the experiment and the contraindications of MRI that were discussed. They signed the consent form before the experiment. They were compensated with a cheque of 30 euros for their participation.

We had to exclude data from 1 adolescent and 5 children. Two of them were discarded because of excessive head movements, 2 had poor eye tracking pupil signal, 1 completed only 4 runs, 1 got anxious in the scanner. Thus, for the reported analyses we included data from 19 adults, aged between 24 and 40 years (mean age = 27.3 ± 5.1 standard deviation, 47.4% female), 19 adolescents aged between 12 and 17 years (mean age 14.1 ± 1.4, 37% female), and 14 children aged between 8 and 11 years (mean age 9.9 ± 0.9, 57% female).

### Visual Stimulation and Eye Tracking

The experiment was programmed using a dedicated software developed under the LabVIEW environment (2017, version 17.0, National Instruments). This software, using parallel programming, allowed synchronizing stimulus display with eye tracking and fMRI recording. The visual stimuli were displayed on a semitransparent screen with a resolution of 1280 × 1024 pixels, placed at the rear of the scanner bore that subjects could view thanks to a mirror attached to the MRI coil just above their eyes. The eye position of the left eye was recorded using an Eyelink 1000 Plus Long Range Mount (SR Research Ltd, Mississauga, Ontario, Canada), using corneal reflection and pupil tracking. The temporal resolution of the eye tracker was 1000 Hz. At the beginning of the session, we carried out a 5-point gaze-calibration. Before each run, the spatial accuracy of the eye tracker was validated against the same 5 points and the system was recalibrated if the worst point error was greater than 1.5° or if the average error exceeded 1.0°.

### Stimuli

As in Morand et al. (2010), we presented 12 images of Caucasian faces with neutral expressions (6 male and 6 female) cropped into an oval shape, as well as 12 images of cars. Both categories of visual stimuli (230 × 230 pixels, 4° visual angle) were gray-scaled photographs on a gray background evened out for luminance, contrast, and spatial frequency, to diminish differences in local low-level visual properties.

### Experimental Design

**Figure 1:**
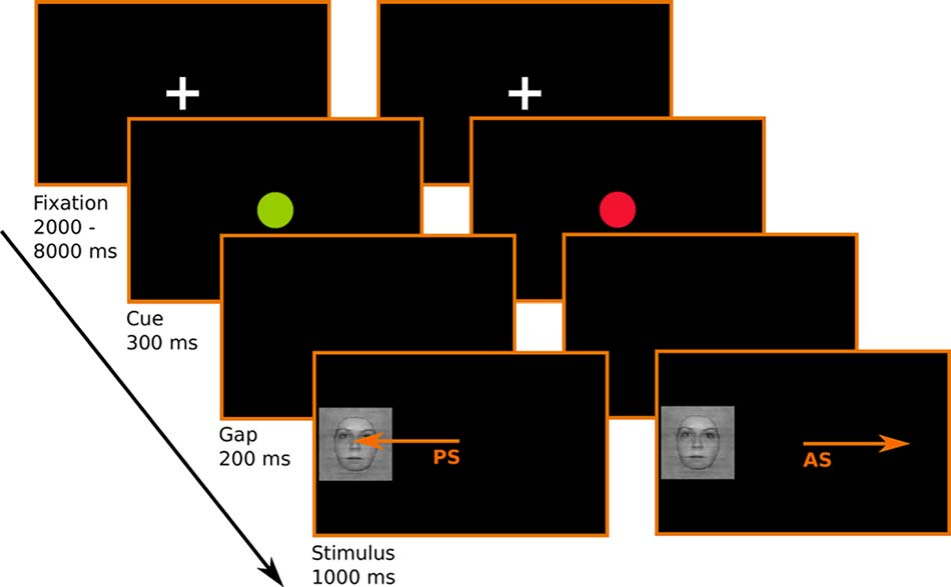
Schematic representation of the antisaccade task. Participants are instructed to look toward the appearing visual stimulus (i.e., produce a prosaccades, PS) when the visual cue following the fixation cross is a green dot. They are instructed to look to the opposite side of the appearing visual stimulus (i.e., produce an antisaccade, AS) when the visual cue following the fixation cross is a red dot.

We implemented an event-related design, mixing pro and antisaccades to faces and cars. Figure 1 represents the time course of the task. Each trial started with a fixation cross displayed for periods ranging from 2000 to 8000 ms (sampled on an exponential distribution, Hagberg et al. 2001), to introduce some jitter between trials onset and thereby improve design efficiency. A visual cue then appeared for 340 ms on the screen. The cue was a central disc (54 × 54 pixels, 1° visual angle) whose color (red or green) defined the nature of the saccadic task that should follow. A blank screen lasting 200 ms marked the transition between the visual cue (central disc) and the visual target stimulus (peripheral image) that was presented 10° to the left or 10° to the right of the screen center for 1000 ms. A green cue prompted the participants to look toward the appearing stimulus (prosaccade) and a red cue signaled that participants should look to the opposite side (to the mirror location) of the appearing stimulus (antisaccade). Participants were instructed to execute pro and antisaccades as quickly as possible. The experiment consisted of six 4-min long runs. Each run comprised 20 face and 20 car stimuli (40 trials in total) with 50% pro and 50% antisaccade instructions, randomized to the left and right hemi-field. We determined 6 different schedules (one for each run) using optseq2 (version 2.0, available at http://surfer.nmr.mgh.harvard.edu/optseq), optimizing the order and timing of the 8 conditions (anti- or prosaccades toward faces or cars to the left or right side of the hemi field). The order of the 6 different schedules was counterbalanced across participants. We explained the task to the participants outside the scanner and had them perform a 2-min long practice session inside the scanner before the experiment started to ensure that the instruction was properly understood. Participants took breaks varying from a few seconds up to 2 min between runs, during which we reminded them of the instructions.

### fMRI Data Acquisition

Brain images were acquired at the Center for MRI of the neurosciences institute of La Timone (http://irmf.int.univ-amu.fr) using a 3 T Siemens Prisma scanner (Siemens, Erlangen, Germany) and a 64-channel head coil. We first acquired a high-resolution *T*_1_-weighted structural image (192 slices, time repetition/time echo [TR/TE] = 2300/2.98 ms, flip angle = 9, voxel size = 1 × 1 × 1 mm^3^, slice thickness = 1 mm, phase encoding direction: i-). To estimate magnetic field inhomogeneity and correct for this during functional data preprocessing, we also acquired a pair of images with opposite phase encoding directions (2 × 54 slices, TR/TE = 7060/59 ms, flip angle = 90°, voxel size = 2.5 × 2.5 × 2.5 mm^3^, slice thickness = 2.5 mm). Then, functional blood oxygen level–dependent (BOLD) images were collected using an echo-planar imaging sequence (54 slices, TR/TE = 1224/30 ms, flip angle: 66°, voxel size = 2.5 × 2.5 × 2.5 mm^3,^ slice thickness = 2.5 mm, field of view = 210 × 210 mm^2^, phase encoding direction: j-, multiband factor = 3, 190 volumes per run).

### Gaze Data Analysis

Gaze parameters were identified with the Eyelink Dataviewer Software (SR Research Ltd, Mississauga, Ontario, Canada), using velocity and acceleration thresholds of 35°/s and 9500°/s^2^, respectively, for saccade detection.

For each trial, the first saccade after stimulus onset that fulfilled the following criteria was analyzed: 1) The saccade latency was longer than 80 ms, as shorter latencies were likely to reflect anticipatory response (Wenban-Smith and Findlay 1991), 2) the saccade amplitude was greater than 2° of visual angle, 3) the saccade direction was clearly classified as “left” or “right.” If this saccade was in the correct direction (toward the target for prosaccade trials and in the opposite direction for antisaccades trials), the trial was classified as correct. If not it was classified as error. When no saccade could be detected, which could be due to no response or eye blink, the trial was classified as “null” trial. In children, adolescents, and adults, respectively, 2.83%, 2.79%, and 1.82% of the trials were considered null trials.

### fMRI Analysis

#### Preprocessing

Preprocessing was done using Feat version 6.00 of FSL. Head motion was computed and corrected within runs using MCFLIRT. Subjects who showed a mean displacement over 2 mm were excluded from the analysis (*n* = 2). For subjects with at least one volume relative to the preceding volume with motion over 2 mm, we applied fsl_motion_outliers (*n* = 9) (Power et al. 2012, 2015). This technique creates a confound matrix to remove the effect of these volumes on the regressors of interest in the general linear model (GLM). Images were unwrapped for distortion correction using *Topup* (Andersson et al. 2003) then the brain was extracted using BET. A Gaussian kernel of 5 mm full-width half maximum was applied to smooth the data. We also computed the nonlinear transformation matrices using FNIRT (Andersson et al. 2007), registering the *T*_1_ anatomical image of each subject to the 2-mm MNI atlas, to apply them to the individual results map and conduct group analyses.

#### Univariate ROI Analysis

##### ROI definition

To look more specifically at the stimulus effect in regions commonly involved in antisaccades and in regions commonly involved in face perception, we used a region of interest approach. Using GingerALE version 3.0.2 (http://brainmap.org/ale/), we defined regions pertaining to the “face perception network” or the “antisaccade network” by computing significant concordant activity in 79 studies, 91% of them conducted in adults, contrasting viewing faces with viewing objects and 13 studies, all conducted in adults, contrasting performing antisaccades to performing prosaccades (references can be found in Supplementary Material). Studies were identified using the application Sleuth (version 3.0.3 http://brainmap.org/sleuth/). The “face network” comprises of left and right fusiform cortex, left and right amygdala, right precentral gyrus, and the right insula. The antisaccade network comprises of left and right middle frontal cortex corresponding to the FEFs, left and right SPL (SPL including IPS), anterior cingulate cortex (ACC), and the right supramarginal gyrus (rSMG). These regions of interest (ROIs) were registered to each subject’s brain using the inverse of the transform matrix computed during preprocessing (see above).

##### Analyses

We conducted analyses at each voxel using the GLM. At the run level, we modeled the BOLD response to correct saccades as a boxcar function featuring events starting at target onsets and lasting 1 s, convolved with a double-gamma function representing the hemodynamic response. We built one separate regressor (explanatory variable) for each condition: antisaccade away from face, antisaccade away from car, prosaccade toward face, and prosaccade toward car. We also modeled 2 additional regressors: errors and null trials. We added the first temporal derivative of each of these original waveforms to account for potential misspecification of the response timing. Only the parameter estimates for the canonical explanatory variables were taken to higher level contrast analyses. Before estimating the model, we applied a high-pass temporal filter to remove low-frequency drifts.

We then averaged the results across runs, yielding one map of mean parameter estimates per participant for each of the 4 conditions. For each participant, we computed the following contrasts of interest (Student’s *t*-test at each voxel): 1) for the task effect: anti versus pro: (antisaccade face + antisaccade car) − (prosaccade face + prosaccade car); 2) for the stimulus effect, since visual stimulation is very different for pro and antisaccades we considered only the prosaccades (for which the stimulus is foveated): proFace versus proCar: (prosaccade face − prosaccade car); 3) for the interaction, we tested for higher task effect for face stimuli: (antisaccade face − prosaccade face) − (antisaccade car − prosaccade car). *T* statistics were converted into Z values.

For each of these 3 contrasts, we extracted from within each ROI in each individual the peak statistical value (Zmax). These values were compared across age-groups using analyses of variance (ANOVAs). We also computed Pearson’s correlations between these values and performance (percentage of antisaccades direction errors and correct antisaccades reaction times) to gain more insight into the brain–behavior relationship. All these analyses were conducted in the R implementation of Jasp (Computer software JASP Version 0.14.1; 2020). *P* values were corrected for multiple comparisons across the different ROIs using the false discovery rate method (FDR; Benjamini and Hochberg 1995).

#### Additional Univariate Analyses

To explore main effects and interactions beyond our main hypotheses, we also entered the individual contrast maps in a voxelwise group analysis using a mixed-model approach with Flame (Woolrich et al. 2004). We examined within-group (children, adolescents, and adults) averaged effects and across group differences. Individual global performance scores (number of correct responses) were entered as covariate to account for group differences in number of correct trials entered in the analyses (note that results changed only marginally without this). The resulting Z (Gaussianised Student’s *t*) statistic images were thresholded using clusters determined by Z > 2.3 and a (corrected) cluster significance threshold of *P* = 0.05 (Worsley 2001).

In addition to the contrasts of interest aforementioned, we also computed the reverse contrasts: pro versus anti (prosaccade face + prosaccade car) − (antisaccade face + antisaccade car); proCar versus proFace: (prosaccade car − prosaccade face); negative interaction: (antisaccade car − prosaccade car) − (antisaccade face − prosaccade face).

#### Multivariate Analysis

We performed MVPA with linear support vector machines (SVMs) as implemented in Nilearn for Python 3.7 (Abraham et al. 2014). We asked whether experimental conditions could be decoded from patterns of activity across voxels of each of our ROIs.

For this analysis, we fitted a new GLM at each voxel using each individual trial as a separate regressor (convolved with the hemodynamic response function). We then trained a linear SVM classifier on the patterns of each condition of interest (antisaccade away from face, antisaccade away from car, prosaccade toward face, and prosaccade toward car) in 4 runs and tested its accuracy to correctly classify the patterns in the 2 remaining runs. Participants’ datasets were imbalanced due to response errors and eye tracker signal loss. To obtain unbiased model predictions, that is, to prevent biasing recognition in favor of the most numerous classes that may result from these imbalanced datasets, we performed bootstrap on our data, repeated 100 times. For each participant, 100 virtual datasets of maps of parameter estimates, each consisting of 6 runs of 40 trials, were created to train and test the linear SVM classifier in all possible combinations of train and test sets. An averaged percent correct classification score per ROI was then computed for each participant from the 6-fold cross-validation per bootstrap. Within each age-group, we compared these values to chance level using *t*-tests. To test for group differences, we submitted individual percent correct decoding score to a 1-way ANOVA with age-group as between-subject factor, in each ROI separately. To further examine the age-group differences, we performed post hoc pairwise *t*-test in each region separately and used FDR to correct for multiple comparisons.

We were interested in the task representation, the stimulus representation, and their interaction. For the task, we trained the classifier on classifying antisaccades as opposed to prosaccades. For the stimulus categorization, we trained the classifier on decoding prosaccades to faces (proFace) from prosaccades to cars (proCar). To question to what extent the stimulus category could impact task representation, at the level of regional activity patterns, we trained the classifier on decoding antisaccades from prosaccades in the face and car conditions separately and tested its decoding accuracy for anti- and prosaccades on the opposite stimulus category (i.e., train on antiFace vs. proFace and test on antiCar vs. proCar or vice versa). Absence of generalization across stimulus categories, that is, at-chance decoding of antisaccades versus prosaccades in the car condition when the classifier was trained on face stimuli or vice versa, would indicate such an interaction. Successful generalization across stimulus category, on the other hand, would suggest similar task regional representation and thus a lack of interaction.

## Results

### Behavioral Results

For correct trials, prosaccade reaction time was on average 211.6 ± 77.7 ms in children, 175.5 ± 48.3 in adolescents, and 178.9 ± 38 ms in adults. It was significantly different between age-groups (*F*_2,49_ *=* 4.8, *P =* 0.01). These differences in reaction time, of the order of maximum 33 ms are unlikely to contribute to detectable changes in the BOLD response, for which we considered 1-s long events and some flexibility in delay is accounted for by adding the temporal derivative. The stimulus effect and the interaction age-group × image category, however, was not significant (*F*_1,49_ *=* 2.23, *P =* 0.1 and *F*_2,49_ *=* 0.35, *P =* 0.7, respectively).

Correct antisaccades mean reaction time was also significantly different between age-groups (*F*_2,49_ *=* 12.09, *P =* 5.4 × 10^−5^), with an average of 273.8 ± 103.1 ms in children, 215.5 ± 60.5 ms in adolescents, and 220.8 ± 53.3 in adults. Image category did not have a significant effect on antisaccade reaction time (*F*_1,49_ = 2.23, *P =* 0.1). In addition, the age-group × image category interaction was not significant (*F*_2,49_ *=* 0.72, *P =* 0.49).

**Figure 2:**
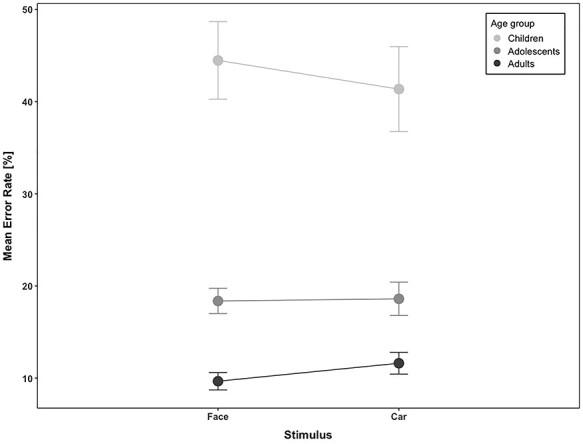
Antisaccade error rate as a function of stimulus category (face and car) in children, adolescents, and adults. Bars indicate standard errors of the mean (SEM).

The prosaccade error rate was overall very small (4%), and thus not further analyzed. The antisaccade error rate was 22% over all subjects across the 3 age-groups and image categories and decreased with age (antisaccade error rate in children = 42.93%; adolescents = 18.49%; adults = 10.64%; *F*_2,49_ *=* 49.4, *P =* 1.8 *E-*12; see Fig. 2). The effect of image category and the interaction between age-group and image category, however, did not yield significant results (*F*_1,49_ *=* 0.13, *P =* 0.7 and *F*_2,49_ = 2.82, *P =* 0.06, respectively).

### Univariate fMRI Results

#### ROI Analyses

**Figure 3:**
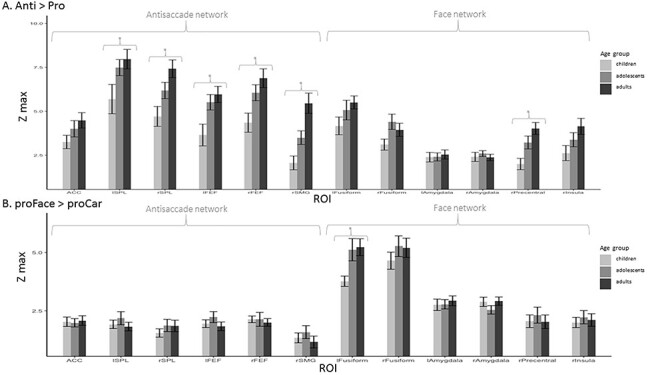
Group-averaged Zmax in all ROIs in children, adolescents, and adults. Bars indicate SEM. (*A*) For the contrast antisaccades > prosaccades. (*B*) For the contrast prosaccades to face > prosaccade to car. Significant differences between groups (*P* < 0.05) are indicated by ^*^.

We were mostly interested in task-related activity, stimulus-related activity, and in the interaction (differences between task-related activity for faces and for cars, respectively) (Fig. 3).

To assess to what extent the amplitude of the task effect changes with age, we compared the average maximal Z statistics (in the contrast anti > pro), in each ROI between age-groups. We found a main effect of age-group in the left and right FEF (*F*_2,49_ *=* 5.4, *P =* 0.007 and *F*_2,49_ = 5.8, *P =* 0.006, respectively), left and right SPL (*F*_2,49_ *=* 3.6, *P =* 0.03 and *F*_2,49_ = 6.6, *P =* 0.003, respectively), right precentral gyrus (*F*_2,49_ = 7.6, *P =* 0.001), and in the rSMG (*F*_2,49_ = 12.2, *P <* 0.001; see Fig. 3*A*). We performed post hoc pairwise *t*-test in these regions to explore group differences in Zmax. After correcting for multiple comparisons, we reported significantly lower Zmax in children compared to adults in the left and right FEF (*t*_31_ = 3.05, *P =* 0.02 and *t*_31_ = 3.24, *P =* 0.02, respectively), in the right SPL (*t*_31_ = 3.55, *P =* 0.01), in the rSMG (*t*_31_ = 4.26, *P <* 0.001), and in the right precentral gyrus (*t*_31_ = 4.14, *P =* 0.004). Zmax was higher in adults than adolescents in the rSMG (*t*_31_ = 3.00, *P =* 0.008). There was no significant difference between adolescents and children.

For stimulus-related activity (proFace vs. proCar), we found a main effect of age-group in the left fusiform cortex (*F*_2,49_ = 3.8, *P =* 0.03), with higher values in adults and adolescents compared to children (see Fig. 3*B*).

As for the task–stimulus interaction, for which the Z values were very low, we did not find any effect of age.

To get a deeper insight into the mechanisms underlying the task-related BOLD signal changes, we examined correlations between Zmax in ROIs of the antisaccade network and behavioral markers of performance, namely percentage of direction errors and saccadic reaction time for correct antisaccades, which reflects difficulty in performing the task. We observed significant negative correlations in FEF, SPL, and SMG (see Supplementary Table 1). This relationship persisted even when factoring out age in bilateral FEF and SPL (although not when correcting for multiple testing in the left SPL), indicating that this association in the whole group was not due to global age-differences in performance but was rather related to the level of performance in the task: Participants who succeeded better in the task also exhibited more activity in the main areas of the antisaccade network.

When looking at the relationship between brain activity and behavior separately for cars and faces, most of the correlations were higher and more significant for faces, especially for saccadic reaction times. To test which correlations were best supported by the data, we compared the correlation models in a Bayesian framework (using R as implemented in JASP). For all regions, there was more support for a (negative) correlation between percentage of errors or mean antisaccades reaction times for trials with a face than a car stimulus (Supplementary Table 2). Note, however, that this comparison is qualitative and that the models are estimated on different data (hence against different null models).

#### Whole Brain Analyses

**Figure 4:**
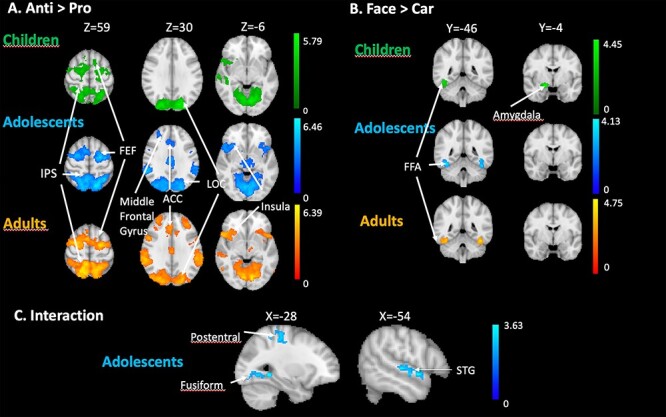
(*A*) Whole brain univariate results for the production of correct antisaccades (anti vs. pro) in children, adolescents, and adults. (*B*) Whole brain univariate results for viewing faces (proFace vs. proCar) in children, adolescents, and adults. (*C*) Whole brain univariate results for the task × stimulus interaction in adolescents. The contrasts are displayed at a threshold using clusters determined by Z > 2.3 and a (corrected) cluster significance threshold of *P* = 0.05.

To get a more complete picture of age-related task- and stimulus effects, we performed an additional whole brain analysis. Figure 4*A–C* depicts above threshold activation loci in the 3 contrasts of interest: anti versus pro, proFace versus proCar, and task-stimulus interaction, respectively. Table 1 contains summarized information of activation peaks and group differences for these contrasts as well as for their opposite (pro vs. anti and proCar vs. proFace). Z (Gaussianised T) statistic images were thresholded using clusters determined by Z > 2.3 and a (corrected) cluster significance threshold of *P* = 0.05. Unthresholded Z-maps have been uploaded to Neurovault (https://neurovault.org/collections/YMAMCKCW/).

**Table 1:** Whole brain univariate analysis results

**A. Antisaccades > prosaccades**								
			**MNI coordinates**					
**Group**	**Voxels**	**Zmax**	***x***		***y***	***z***	**Approximate location**	
**Children**	16 520	5.41 *5.04*	30 *−20*		−78 *−66*	16 *−12*	LOC *L occipital fusiform*	
	4266	5.35	28		−8	48	R precentral gyrus	
	923	3.63	10		−20	6	R Thalamus	
	712	3.74	64		−44	26	R SMG	
	461	3.77	40		12	−2	R Insula	
	423	3.71	−18		44	26	L SFG	
**Ado**	31 551	6.21 *5.95* *5.58*	−12 *−14* *−24*		−74 *−68* *−56*	46 *60* *66*	Precuneus cortex *LOC* *L SPL*	
	7587	5.89 *5.82* *5.59*	−24 *−32* *−28*		−6 *−10* *−4*	58 *50* *52*	L SFG *L Precentral gyrus* *L MFG*	
	4428	4.36	10		−6	20	R caudate	
	974	4.13 *4.12* *4.1*	34 *50* *−12*		22 *24* *−6*	10 *−2* *14*	R Insula *R IFG* *Thalamus*	
	469	3.6	30		28	38	R MFG	
**Adult**	34 032	6.39 *6.17* *6* *5.95*	12 *−10* *52* *16*		−66 *−80* *−72* *−60*	60 *−4* *16* *58*	R + L Precuneus cortex *Lingual gyrus* *LOC* *SPL*	
	16 049	6.06 *5.74*	32 *−24*		−2 *−6*	52 *58*	*MFG* *SFG*	
	607	4.1	−36		38	32	L MFG	
**Child > ado**	787	3.23	−12		56	38	L SFG	
	714	3.99	30		−78	16	R LOC	
	505	3.46	−16		−88	10	R + L Occipital pole	
**Ado > child**	514	3.21	−60		−38	48	L SMG	
**Adults > chil**	1283	4.01	−62		−28	36	L SMG	
**B**. Prosaccades to face > prosaccades to car								
**Group**	**Voxels**	**Zmax**	***x***	***y***	***z***	**Approximate location**		
**Children**	881	4.44	42	−52	−28	R temporal occipital fusiform		
	404	4.34	18	−6	−14	R Amygdala		
	377	3.96	−42	−56	−22	L temporal occipital fusiform		
**Ado**	595	4.46	42	−50	−20	R temporal occipital fusiform		
	529	3.86	−42	−44	−26	L temporal fusiform		
**Adults**	635	4.38	−42	−52	−22	L temporal occipital fusiform		
	490	4.8	42	−60	−20	R temporal occipital fusiform		
**Ado > adults**	616	3.64	−46	28	26	L MFG		
**Adults > ado**	553	3.37	60	−2	10	R precentral		
**C. Interaction: (antiFace–proFace) > (proFace–proCar)**								
				**Coordinates**				
**Group**	**Voxels**	**Zmax**		***x***	***y***	***z***	**Approximate location**	
**Adolescents**	689	3.52		36	−72	10	R LOC	
	498	3.56		−24	−66	−6	L occipital fusiform gyrus	
	474	3.63		−54	0	−6	L STG	
	453	3.14		2	−28	52	SMA	
	418	3.23		−32	−28	62	Postcentral gyrus	
**D. Prosaccades > antisaccades**								
**Group**	**Voxels**	**Zmax**	***x***	***y***	***Z***	**Approximate location**		
**Children**	2591	5.74	34	−92	0	R LOC		
	2101	5.67	−36	−94	−6	L LOC		
**Adolescents**	4933	6.92	26	−90	−16	R occipital fusiform gyrus, R LOC		
	3085	5.68	−32	−90	−10	L occipital fusiform cortex, LLOC		
	2209	4.17	−4	66	22	Frontal pole		
	1629	4.46	−4	−24	60	L Precentral gyrus		
	1338	4.28	−52	30	16	L IFG		
	906	4.59	−10	32	−12	Frontal medial cortex		
	538	4.02	−36	−14	−28	L temporal fusiform cortex		
	431	4.52	36	34	−10	R frontal pole		
**Adults**	11 059	7.03	34	−44	−20	R temporal occipital fusiform		
	1489	4.12	−4	34	−22	Frontal medial cortex		
	1232	3.98	−12	56	36	Frontal pole		
	1122	4.3	68	−4	26	R postcentral gyrus, R precentral		
	1115	4.28	−52	−20	58	L postcentral gyrus		
	752	3.91	−12	−44	38	Cingulate gyrus		
**Child > ado**	514	3.21	−60	−38	48	L SMG		
**Child > adults**	1283	4.01	−62	−28	36	L SMG		
**Adults > child**	787	3.23	−12	56	38	L SFG, L MFG		
	714	3.99	30	−78	16	R LOC		
	505	3.46	−16	−88	10	L LOC		
**E. Prosaccades to car > prosaccades to faces**								
**Group**	**Voxels**	**Zmax**	***x***	***y***	***Z***	**Approximate location**		
**Adolescents**	711	3.51	66	−26	10	R STG		
	386	3.37	−22	−30	52	L central sulcus		
	355	3.69	36	−72	12	R LOC		
**Adults**	648	3.71	30	−82	4	R LOC		
	447	3.73	−50	24	30	L MFG		
**Ado > adults**	553	3.37	60	−2	10	R inferior precentral G		
**Adults > ado**	616	3.64	−46	28	26	L MFG		

Z (Gaussianised T) statistic images were thresholded using clusters determined by Z > 2.3 and a (corrected) cluster significance threshold of *P* = 0.05. Coordinates for up to 4 peaks per cluster are presented. Subpeaks Z statistics, MNI coordinates, and approximate locations are presented in italic.

As depicted in Figure 4*A*, and confirming the ROI analysis, children, adolescents, and adults showed activity at the level of the FEF and IPS when producing antisaccades (anti- vs. procontrast). In addition, we observed significant antisaccade-related activity in all groups in LOC and insula. Both adolescents and adults’ groups showed activity at the level of the ACC and the middle frontal gyrus (MFG). As for face-related activity (proFace vs. proCar), the 3 groups showed activity in the fusiform cortex, bilaterally. Children showed significant activity in the amygdala (Fig. 4*B*). The task × stimulus interaction (antiFace–proFace vs. antiCar–proCar) was significant in adolescents in the postcentral gyrus and in the fusiform cortex (Fig. 4*C*), in the left LOC, left precentral gyrus, and right superior temporal gyrus. In these regions, the difference of activity in anti- versus prosaccades was greater when the stimulus was a face compared to a car. Changing the threshold to *P* < 0.001 uncorrected revealed an interaction in the adults group as well, but in different regions than those observed in the adolescents group. These were located in the right superior and middle frontal gyri, paracingulate gyrus, and bilateral LOC (although not overlapping with the region observed in adolescents) and SPL. In children, even lowering the threshold did not reveal interaction between task and stimulus in any part of the brain.

### Multivariate Analyses Results

**Figure 5:**
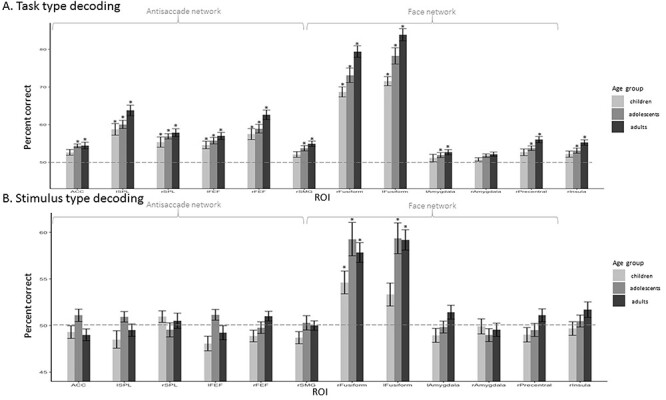
ROI-based MVPA: mean percentage decoding accuracy in children, adolescents, and adults. SVM classification accuracy for saccade category (task) detection was averaged per age-group per region. Statistical significance was assessed with 1-sample *t*-test against 50% chance and corrected for multiple comparisons using the FDR method. Above chance statistical significance (*P* < 0.05) is represented by ^*^. Significant differences between groups of *P* < 0.05 are represented by ^*^. (*A*) Saccade type (task) decoding. (*B*) Stimulus type (category) decoding.

Figure 5 depicts decoding accuracies in each ROI for each age-group. We found above chance decoding of anti- versus prosaccades in all regions involved in antisaccades (ACC, left and right SPL, left and right FEF, and in the rSMG) in all age-groups. We also found above chance decoding in the 3 groups in the left and right fusiform cortex. In addition, above chance accuracy was found in the left amygdala, right precentral, and right insula in both adolescents and adults, as well as in the right amygdala in adults (see Fig. 5*A* and Supplementary Table 3). To test for group differences, we submitted individual percent correct decoding scores to a 1-way ANOVA with age-group as between subject factor, in each ROI separately, and we used FDR to correct for multiple comparisons. In the antisaccade network, we found a main effect of group in the right FEF (*F*_2,49_ = 4.268, *P =* 0.02). We also observed a group effect in the fusiform gyrus (right: *F*_2,49_ = 9.866, *P =* 0.0003; left: *F*_2,49_ = 10.84, *P =* 0.0001), the right precentral (*F*_2,49_ = 4.813, *P =* 0.01), and the right insula (*F*_2,49_ = 4.555, *P =* 0.02) from the face network. To further examine the differences between age-groups, we performed post hoc pairwise *t*-tests in each region separately, when the ANOVA had revealed a significant main effect. These tests revealed better decoding performance in the right FEF in adults compared to children (*t*_31_ = 2.66, *P =* 0.01) and in adults compared to adolescents (*t*_36_ = 2.16, *P =* 0.04). In the right and left fusiform, right precentral, and right insula, better decoding performance was found in adults compared to children (*t*_31_ = 5.28, *P =* 9.65E-06; *t*_31_ = 6.07, *P =* 1.07E-06; *t*_31_ = 2.75, *P =* 0.01; *t*_31_ = 2.83, *P =* 0.009, respectively) and in adults compared to adolescents (*t*_36_ = 2.56, *P =* 0.01; *t*_36_ = 2.08, *P =* 0.05; *t*_36_ = 2.22, *P =* 0.03; *t*_36_ = 2.17, *P =* 0.04). In the left fusiform, we also found better decoding performance in adolescents compared to children (*t*_31_ = 2.71; *P =* 0.011).

For stimulus category decoding, we found above-chance levels of decoding in the left and right fusiform gyri in all age-groups except in children in the left fusiform gyrus (see Supplementary Table 4). The ANOVA did not reveal any significant effect of age-group on correct decoding after correcting for multiple comparisons.

In all the regions where the anti/prosaccade decoding was significantly above chance (see above and Fig. 5), we observed also a significant decoding (*t*-tests corrected with FDR methods, all *P*s < 0.04) across categories, that is, when the classifier was trained on distinguishing patterns related to anti- versus prosaccades on trials of one stimulus category and tested on the other stimulus category. There was indeed no difference in performance between decoding within the trained category and across categories. This generalization indicates that the information carried in the pattern of activity of these regions that distinguishes antisaccades from prosaccades does not depend on the stimulus.

## Discussion

We investigated the influence of a social stimulus on inhibitory control, and the brain regions involved in this process during late childhood and adolescence. We addressed this issue by using an antisaccade task with social and nonsocial stimuli during fMRI. We carried out univariate analyses to investigate differences in activity in ROIs, defined independently as associated with antisaccades or with face perception, and were complemented by whole brain analyses to further explore potential differences in activity outside these networks. Finally, we conducted MVPA to capture the strength of the representations of task and stimulus in the ROIs in the 3 age-groups as well as potential differences between age-groups. We replicated previous findings of the immaturity of inhibitory control in children and adolescents: decreased reaction time and inhibitory errors in the antisaccade task with age; increased engagement of regions involved in producing antisaccades with age. Similarly, face-related activity in the FFA was observed in all age-groups, but its magnitude increased from childhood to adulthood. Adolescents were the only age-group that showed a modulation of task-related activity by stimulus category that remained significant after correcting for multiple comparisons: Antisaccades-related activity was significantly increased for faces compared to cars in the postcentral and precentral gyri, in the STG, and in the fusiform cortex. We discuss these results in relation to previous literature on the maturation of antisaccade and social perception, focusing more specifically on adolescence.

### Age-Related Changes in Inhibitory Control

The observed decrease of reaction times of correct antisaccades and antisaccades direction error rates with age most likely reflects an improvement in inhibitory control. It replicates previous developmental studies on antisaccades (Fischer et al. 1997; Munoz et al. 1998; Luna et al. 2001; Luna and Sweeney 2004; for review Karatekin 2007). It is concordant with a large corpus of developmental studies that showed that cognitive control improves from childhood to adulthood (Ridderinkhof and Van Der Molen 1997; Cepeda et al. 2001; Bunge et al. 2002; Davidson et al. 2006; Rubia et al. 2006; rev in Diamond 2013).

Our brain imaging analyses confirmed that the nodes of basic oculomotor circuitry—namely FEF and parietal cortex—are already identified at a young age and that they show higher activity for antisaccades compared to prosaccades (as in for instance Luna et al. 2001). Their activity was correlated with task performance: Individuals who performed the task better (i.e., faster responses and with fewer errors) also showed higher activity in this network. This indicates that the identified change in BOLD signal was associated with the level of performance in the task, and thus to the correct inhibition and reprogramming of a saccade, consistent also with previous reports in adults (Connolly et al. 2005) and children and adolescents (Alahyane et al. 2014; Quach et al. 2020). The comparison of peaks in contrasts of parameter estimates (Zmax) between groups in the antisaccade network revealed stronger activity in FEF and SPL with age. This implies that, although all 3 age-groups recruit the same regions, the level of antisaccade-specific activity in these regions increases with age consistent with previous reports (Luna et al. 2001; Geier et al. 2010; Alahyane et al. 2014; Quach et al. 2020). These results, however, are at odds with other studies that do not find developmental differences in these oculomotor regions (Velanova et al. 2008; Padmanabhan et al. 2011; Ordaz et al. 2013). Most of these, however, report antisaccade activity relative to baseline thus mixing both inhibition and execution. Only Luna et al. (2001), like we do in the present study, report the contrast between anti- and prosaccades, hence removing the effect of common sensorimotor processing for both prosaccades and antisaccades and isolating the effects of inhibition and reprogramming. They, like we do here, observe an increased activity in oculomotor regions. In addition, these studies (except for Alahyane et al. 2014) used blocks of antisaccades and blocks of prosaccades. As mixing antisaccades and prosaccades trials increases task difficulty (Los 1991; Cherkasova et al. 2002), the age-related increase in recruitment of frontoparietal regions may partly be due to increased attentional load. This goes in line with results from analyses of cue-related activity only that show an increased recruitment of these regions with age only for this anticipatory phase of antisaccades when participants have to adapt their task set (Geier et al. 2010, Alahyane et al. 2014). Moreover, MVPA, which normalized overall local activity, revealed that although the task (i.e., whether participants performed a pro or an antisaccade) could be decoded successfully in frontoparietal regions in all age-groups, the decoding accuracy was higher for adults than younger participants in the right FEF. This suggests that in adolescence and childhood, the task representation in frontal regions is still not as robust as it is in adults. In the dACC, another region often identified in antisaccades studies, we observed significant antisaccades-related activity only in adolescents and adults in the whole brain univariate analysis. Moreover, the ability to decode task from activity patterns was significant only in adolescents and adults (although at a relatively low level) and not in children. Velanova et al. (2008) also identified an increased involvement of dACC with age. The dACC is known to be involved in error monitoring and feedback (Braver et al. 2001; Rubia et al. 2007). Since the involvement of the dACC is often associated with feedback signaling, its increased involvement with age could be due to less feedback signaling in younger age-groups and immature error regulation, as proposed in Velanova et al. (2008). This, however, is inconsistent with the study conducted by Alahyane et al. (2014) that reported an increased involvement of the dACC with age for the task preparatory phase, but not during the task itself when error monitoring is potentially occurring. Because we did not have enough trials, we could only observe a more general immaturity in the involvement of this region in our task requiring inhibitory control. It would be interesting for future studies, however, to directly compare to directly compare erroneous to correct antisaccades trials with regards to dACC involvement. Another region, that is, sometimes reported in developmental antisaccade studies is the MFG (or dorsolateral prefrontal cortex), but results have been controversial. Some studies report a decrease of MFG activation between childhood and adulthood (Velanova et al. 2008; Ordaz et al. 2013). This is interpreted as a decrease in effort to correctly inhibit a response. Other studies, however, report an increase in MFG involvement with age, implying a delayed maturation of the MFG and of the frontal cortex in general (Bunge et al. 2002; Rubia et al. 2007). But even in adults’ populations, the MFG is inconsistently identified during antisaccades, as shown by the fact that this region does not show up in the meta-analysis we performed to define the ROIs of the antisaccade network. It is most probably, like the ACC, involved in task-set selection and thus might not be much engaged in tasks using block designs. In their study, Jarvstad and Gilchrist (2019), who used blocked design to compare saccade choice, inhibition, and selection, only found the MFG and ACC to be involved in the choice contrast, which in their case was the only contrast with high cognitive demand. This once again suggests that the involvement of these regions reflects higher level processes rather than inhibition per se. Our study not only mixes pro and antisaccades, in unpredictable hemifield, but also mixes trials with different stimuli, which for adults, adolescents, and children have probably different values. This may mobilize more resources linked to task monitoring and explain the significant activity in the MFG for trials necessitating inhibition and reprogramming of a saccade. This, however, was observed in adults and adolescents, but not in children. Thus, altogether our data indicate that regions involved in general task monitoring (like ACC and MFG), while very little engaged in children, show already an adult-like activity in adolescents. Activity in oculomotor regions, mainly the FEF in the right hemisphere, however, is still significantly lower in adolescents compared to adults. It would be interesting to evaluate how these results would generalize across other inhibitory control tasks that do not necessitate the oculomotor network.

### Face Processing

As expected, we found activity in children, adolescents, and adults in the FFA when they made a saccade toward a face. In addition, we found a slight increase in Zmax in FFA with age occurring mainly between children and adolescents with both adults and adolescents showing higher Zmax than children, concordant with previous studies (Scherf et al. 2007; Peelen et al. 2009; Golarai et al. 2015). This increased implication with age could reflect the cumulative exposure to faces with age (Cohen Kadosh et al. 2013; Arcaro et al. 2017). Previous behavioral studies point out that children, like adolescents and adults, are attracted early on to faces and spend a significant time looking at them, even though some aspects of social stimulus processing, such as face processing, mental state inference, and responding to peer influence and social evaluation, continue to mature until at least mid-adolescence (as reviewed in Burnett et al. 2011).

### Interaction between Inhibitory Control and Face Processing

Our behavioral findings do not replicate previous reports of increased antisaccades errors for faces compared with cars (Gilchrist and Proske 2006; Morand et al. 2010). This could be due to the limited number of trials leading to reduced statistical power to detect small differences compared with studies conducted outside the scanner. Yet despite this lack of stimulus effect at the behavioral level, we could observe that the correlation between performance and brain activity in regions of the antisaccade network was better supported, in a Bayesian framework analysis, in the data with faces as stimuli, compared with cars. This suggests that the task-related activity is more robustly associated with success in the task, that is, inhibiting a saccade and reprogramming a new one, when the stimulus is a face, hence indicating a special status of social stimuli (at least faces).

Our relatively small number of participants precludes examining these correlations in separate groups, however. In a larger developmental behavioral study (*n* = 139) (Geringswald et al. 2020), we found that although adults and adolescents had more difficulty inhibiting their reflexive saccade to a face than other stimuli, this was not the case for children, implying that the special involuntary attraction to faces would only appear in adolescence. This suggests that despite the lack of apparent effect at the behavioral level, we could still expect differences in the fMRI signal for the interaction between task and stimulus category between our 3 age-groups.

More specifically, we expected to find an interaction between task and stimulus in the antisaccade network for the adolescents and adults participants. This was not the case, that is, in these regions, the antisaccade-prosaccade contrast was not modulated by the stimulus category. This was comforted by the MVPA that showed a generalization of task decoding across stimulus categories: Task could be decoded from patterns of activity in all ROIs whether the classifier had been trained on the same or the other category of stimulus. We only found a significant interaction between the task and the stimulus in adolescents and in areas outside our a priori ROIs: Antisaccade-related activity was higher in the face than in the car conditions at the level of the postcentral gyrus, paracentral sulcus (supplementary motor area/SEF), STG, occipital fusiform gyrus, and LOC. It may not be surprising to find an interaction between task and stimulus in occipital regions like the LOC and the occipital fusiform gyrus since the visual cortex is sensitive to stimulus category and its eccentricity (Malach et al. 2002) and displayed indeed higher activity for cars than for faces. The postcentral gyrus and supplementary motor cortex are more unexpected. Both these regions have, however, been mentioned in previous studies exploring antisaccades, with higher activity related to antisaccades as compared to prosaccades (Brown et al. 2006; Velanova et al. 2008; Jamadar et al. 2013). The fact that we observe these regions more in the face than in the car antisaccade–prosaccade contrast and only in adolescents suggests that they may have a role in adapting inhibitory control to context and that this may depend on individual factors including age. In addition, the involvement of the STG could be due to its role in social information processing (Blakemore 2008; Steinberg 2008). This region is also known to show increased activity related to face processing from childhood to adulthood (Burnett et al. 2011). In adults, a task–stimulus interaction could be observed only without correction for multiple comparisons, but in different regions, including the superior frontal sulcus (as in Salvia et al. 2020), middle frontal and paracingulate gyri, bilateral LOC, and SPL. This set of regions is very similar to what is commonly observed in voluntary covert shifts of attention (Grosbras et al. 2005). Thus, it seems that the differential task-related activity as a function of stimulus might have different meanings in adults and adolescents. In adults, it might reflect the higher attentional demand over the automatic orienting to faces. In adolescents, it could relate to a special treatment of social stimuli. Indeed adolescence is a period that is marked by critical changes in both social and inhibitory functions (Scherf et al. 2007). Investigating the effect of reward on antisaccade performance, several studied reported less errors in rewarded trials as well as a higher engagement of brain networks associated with rewards in adolescents compared to adults (Geier et al 2010; Padmanabham et al; 2011; Hallquist et al 2018). They respectively showed an increased activity along the precentral sulcus (likely corresponding to the FEF) for the preparation of an antisaccade from a rewarded trial and higher ventral striatal activity during rewarded trials. This implies that in adolescents external factors influence performance on the antisaccade task, that is, inhibitory control—as well as brain regions’ engagement. Just like the reward system, the “social perception system” also continues to develop during adolescence and reward and social contexts seem to influence adolescents’ cognition to a higher extent than in children and adults. Thus, we argue that the effect of social cues on brain regions engaged during inhibitory control is different in adolescents from adults and children. The fact that social influence during adolescence is special, could explain that in adolescents compared with adults, the task–stimulus interaction observed in brain activity is stronger and expressed in brain regions possibly more related to social processing.

### Limitations and Future Directions

One limitation of our study is that we compare brain regions for antisaccades in the 3 age-groups knowing that we have less correct antisaccade trials for children than for adolescents and adults. Although this was taken into account in our GLM, it remains a general limitation in developmental fMRI studies where participants are asked to perform a task that is cognitively demanding. Also, as in many developmental studies, we could not use data from all participants due to head motion. Our findings should thus be replicated with a larger sample of participants. Besides, we test the effect of social stimuli on inhibitory control by the presentation of a relatively impoverished stimulus consisting of faces with neutral expressions. Indeed to control for lower level visual stimuli, we presented gray-scaled faces and cars on a gray background. We recognize that more realistic social stimuli with possible increased saliency, like emotional faces, could have yielded increased influence on inhibitory control. It is thus possible that the use of impoverished stimuli might have had a different effect in adolescents for whom faces might have appeared “strange,” children for whom both categories of stimuli might have appeared equally strange and adults who might be less sensible to the image manipulation. It would be interesting to reproduce a similar antisaccade task in which the faces presented are more realistic social cues, or with an affective or salient expression.

In addition, we presented adult faces as social stimuli, hence possibly creating an own-age effect in adults (Ebner and Johnson 2010). It would be interesting to evaluate this effect by conducting a similar study presenting faces from all age-groups and investigating the own-age effect on inhibitory control. Beyond age, it might be interesting to question whether group-membership based on other factors, like ethnicity would affect the specialized effect of faces. For instance, out-group membership based on ethnicity was found to provoke a different automatic imitation response than in-group membership, both behaviorally and in fMRI activity patterns when participants viewed faces belonging to in- or out-group ethnicity (Rauchbauer et al. 2015). In addition, it has to be noted that, given our recruitment channel, mainly through University staff network, our sample is not be representative of the population at large. Indeed all participants came from middle- or high-income families; in addition, although not specifically intended in our recruitment, all participants were Caucasian. As individual factors (like ethnicity; Rauchbauer et al. 2015) and environmental factors (like socio-economic status; Quach et al. 2020; Tooley et al. 2020) can impact both cognitive control and social perception as well as brain organization, it would be interesting to extend this study to a larger population, to test the influence of these factors.

In conclusion, our results extend the existing literature on the development of the antisaccade network and the face network. We confirm that the core nodes are already present at a young age but that the strength of activity and pattern representations continues to increase during adolescence. In addition, we also found an effect of socially relevant stimuli on activity during inhibitory control in brain regions outside our ROIs, implying they are important for social processing. This interaction was observed only in the adolescent brain, endorsing the importance of understanding the adolescent brain as a crucial developmental period for social perception as well as cognitive control.

## Supplementary Material

CerCorCom_afyouni_SI_tgab057

## Notes

We thank Jean-Luc Anton and Julien Sein for assistance for implementing the scanning sequences and protocol; Laure Spieser for her assistance with the MVPA analysis; Marianne Vaugoyeau for helping us with the recruitment of participants and scanning sessions. *Conflict of Interest*: None declared.
