# Supplementary material for: Brain Activity During Antisaccades to Faces in Adolescence
Source: Cereb Cortex Commun. 2021 Sep 24;2(4):tgab057. doi: 10.1093/texcom/tgab057 (PMC8597975; doi:10.1093/texcom/tgab057)
Supplement: CerCorCom_afyouni_SI_tgab057 [file cercorcom_afyouni_si_tgab057.pdf]

Brain activity during antisaccades to faces in adolescence

SUPPLEMENTARY INFORMATION

REFERENCES FOR REGIONS OF INTEREST DEFINITIONS

References included in the meta-analyses to define ROIs in the Antisaccade network and in the Face Network (N=number of healthy participants, age indicated if under 18)

Antisaccade network

Authors & Year	Journal	Title	N (age range)
O'Driscoll et al. 1995	Proceedings of the national academy of sciences	Functional neuroanatomy of antisaccade eye movements investigated with positron emission tomography	10
Paus et al. 1993	Journal of neurophysiology	Role of the human anterior cingulate cortex in the control of oculomotor, manual, and speech responses: a positron emission tomography study	9
Connolly et al. 2000	Journal of neurophysiology	A comparison of frontoparietal fMRI activation during anti-saccades and anti-pointing	7
Kimmig et al. 2001	Experimental brain research	Relationship between saccadic eye movements and cortical activity as measured by fMRI: quantitative and qualitative aspects	15
Matsuda et al. 2004	Psychiatry research: neuroimaging	Functional MRI mapping of brain activation during visually guided saccades and antisaccades: cortical and subcortical networks	21
Sweeney et al. 1996	Journal of neurophysiology	Positron emission tomography study of voluntary saccadic eye movements and spatial working memory	11
Culham et al. 2003	Experimental brain research	Visually guided grasping produces fMRI activation in dorsal but not ventral stream brain areas	10
Ford et al. 2005	Journal of neurophysiology	Neural processes associated with antisaccade task performance investigated with event-related FMRI	10
Chikazoe et al. 2007	Journal of cognitive neuroscience	Activation of right inferior frontal gyrus during response inhibition across response modalities	24
Brown et al. 2006	NeuroImage	Inhibition and generation of saccades: Rapid event-related fMRI of prosaccades, antisaccades, and nogo trials	10
Ettinger et al. 2008	Cerebral cortex	Decomposing the neural correlates of antisaccade eye movements using event-related FMRI	17
Brown et al. 2007	Neuroimage	Isolation of saccade inhibition processes: Rapid event-related fMRI of saccades and nogo trials	11

Collette et al. 2005	Human brain mapping	Exploring the unity and diversity of the neural substrates of executive functioning	37

Faces

Authors & Year	Journal	Title	N (age range)
Hariri et al. 2000	Neuroreport	Modulating emotional responses: effects of a neocortical network on the limbic system	16
Hasson et al. 2002	Neuron	Eccentricity bias as an organizing principle for human high-order object areas	13
Sugiura et al. 2001	Neuroimage	Activation reduction in anterior temporal cortices during repeated recognition of faces of personal acquaintances	7
Gorno-Tempini et al. 2001	Neuroimage	Explicit and incidental facial expression processing: an fMRI study	10
Kesler-West et al. 2001	Cognitive brain research	Neural substrates of facial emotion processing using fMRI	21
Paller et al. 2003	Learning & memory	Neural correlates of person recognition	20
Wright et al. 2002	Neuroreport	Enhanced amygdala responses to emotional versus neutral schematic facial expressions	16
Braver et al. 2001	Neuroimage	Direct comparison of prefrontal cortex regions engaged by working and long-term memory tasks	28
George et al. 1993	J. neuropsychiatry and clinical neuroscience	Brain regions involved in recognizing facial emotion or identity: An oxygen-15 PET study	9
Kringelbach et al. 2003	Neuroimage	Neural correlates of rapid reversal learning in a simple model of human social interaction	9
Williams et al. 2005	Neuroimage	Differential amygdala responses to happy and fearful facial expressions depend on selective attention	13
Bonner-Jackson et al. 2005	Biological psychiatry	The influence of encoding strategy on episodic memory and cortical activity in schizophrenia	26
Pierce et al. 2004	Brain	The brain response to personally familiar faces in autism: Findings of fusiform activity and beyond	10 (16–40 years)
Dalton et al. 2007	Biological psychiatry	Gaze-fixation, brain activation, and amygdala volume in unaffected siblings of individuals with autism	19 (8 - 25 years)
Dolan et al. 1996	Neuroimage	Neural activation during covert processing of positive emotional facial expressions	8

Dolan et al. 2001	Proceedings of the National Academy of Sciences	Crossmodal binding of fear in voice and face	12
Schaefer et al. 2006	Biological psychiatry	Event-related functional magnetic resonance imaging measures of neural activity to positive social stimuli in pre- and post-treatment depression	17
Bird et al. 2006	Neuroimage	Attention does not modulate neural responses to social stimuli in autism spectrum disorders	16
Dapretto et al. 2006	Nature neuroscience	Understanding emotions in others: Mirror neuron dysfunction in children with autism spectrum disorders	9 (12 ± 2 years)
Vuillemier et al. 2001	Neuron	Effects of attention and emotion on face processing in the human brain: An event-related fMRI study	12
Deeley et al. 2007	Biological psychiatry	An event related functional magnetic resonance imaging study of facial emotion processing in Asperger syndrome	18
Denkova et al. 2006	Neuropsychologia	Neural correlates of remembering/knowing famous people: An event-related fMRI study	12
Goekoop et al. 2005	Neuroimage	Raloxifene exposure enhances brain activation during memory performance in healthy elderly males; its possible relevance to behavior	30 (60 to 70 years)
Payer et al. 2008	Drug and alcohol dependence	Differences in cortical activity between methamphetamine-dependent and healthy individuals performing a facial affect matching task	24
Rotshtein et al. 2007	Journal of cognitive neuroscience	Role of features and second-order spatial relations in face discrimination, face recognition, and individual face skills: Behavioral and functional magnetic resonance imaging data	19
Wild et al. 2003	Psychiatry research	Why are smiles contagious? An fMRI study of the interaction between perception of facial affect and facial movements	10
Benuzzi et al. 2007	Brain research bulletin	Processing the socially relevant parts of faces	24
Britton et al. 2006	Neuroimage	Facial expressions and complex IAPS pictures: Common and differential networks	12
Dolcons and McCarthy 2006	Journal of neuroscience	Brain systems mediating cognitive interference by emotional distraction	18
Feusner et al. 2007	Archives of general psychiatry	Visual information processing of faces in body dysmorphic disoder	13
Platek et al. 2006	Human brain mapping	Neural substrates for functionally discriminating self-face from personally familiar faces	12

Holt et al. 2006	Schizophrenia research	Increased medial temporal lobe activation during the passive viewing of emotional and neutral facial expressions in schizophrenia	16
Franz and Ishai 2006	Current biology	Face perception is modulated by sexual preference	40
Leibenluft et al. 2004	Biological psychiatry	Mother's neural activation in response to pictures of their children and other children	20
Nakamura et al. 1998	Neuroreport	Neuroanatomical correlates of the assessment of facial attractiveness	6
O'Dherty et al. 2003	Neuropsychologia	Beauty in a smile: The role of medial orbitofrontal cortex in facial attractiveness	25
Altshuler et al. 2008	Bipolar disorders	Regional brain changes in bipolar I depression: A functional magnetic resonance imaging study	17
Winston et al. 2007	Neuropsychologia	Brain systems for assessing facial attractiveness	28
Blonder et al. 2004	Cognitive brain research	Regional brain responses to faces of humans and dogs	14
Pourtois et al. 2005	Cortex	Perception of facial expressions and voices and of their combination in the human brain	8
Bokde et al. 2008	Psychiatry research	Functional abnormalities of the visual processing system in subjects with mild cognitive impairment: An fMRI study	19 (mean age 66.7)
Devue et al. 2007	Brain research	Here I am: The cortical correlates of visual self-recognition	20
Blasi et al. 2009	Psychiatry research	Changes in prefrontal and amygdala activity during olanzapine treatment in schizophrenia	12
Fleming et al. 2010	Journal of neurophysiology	Effects of category-specific costs on neural systems for perceptual decision-making	19
Victor et al. 2010	Archives of general psychiatry	Relationship between amygdala responses to masked faces and mood state and treatment in major depressive disorder	24
Gilman et al. 2008	Journal of neuroscience	Why we like to drink: A functional magnetic resonance imaging study of the rewarding and anxiolytic effects of alcohol	12
Bertocci et al. 2012	Psychological medicine	Abnormal anterior cingulate cortical activity during emotional n-back task performance distinguishes bipolar from unipolar depressed females	16
Dichter et al. 2012	Journal of autism and developmental disorders	Reward circuitry function in autism during face anticipation and outcomes	20
Kleinhans et al. 2009	American journal of psychiatry	Reduced neural habituation in the amygdala and social impairments in autism spectrum disorders	20

Kleinhans et al. 2010	Neuropsychologia	Association between amygdala response to emotional faces and social anxiety in autism spectrum disorders	29
Uddin et al. 2008	PLoS ONE	Neural basis of self and other representation in Autism: An fMRI study of self-face recognition	12
Vizueta et al. 2012	American journal of psychiatry	Regional fMRI hypoactivation and altered functional connectivity during emotion processing in nonmedicated depressed patients with bipolar II disorder	21
Gentili et al. 2009	Brain Research Bulletin	Beyond amygdala: Default Mode Network activity differs between patients with Social Phobia and healthy controls	7
Greimel et al. 2010	Journal of neural transmission	Development of neural correlates of empathy from childhood to early adulthood: An fMRI study in boys and adult men	47 (8–27 years)
Kleinhans et al. 2008	Brain	Abnormal functional connectivity in autism spectrum disorders during face processing	23
Ramirez-Ruiz et al. 2008	Movement Disorders	Brain response to complex visual stimuli in Parkinson's patients with hallucinations: A functional magnetic resonance imaging study	10
Prochnow et al. 2014	Brain and cognition	Reasoning about the implications of facial expressions: A behavioral and fMRI study on low and high social impact	26
De Greck et al. 2013	Human brain mapping	Altered brain activity during emotional empathy in somatoform disorder	20
Fairhall et al. 2014	Cerebral cortex	Person- and place-selective neural substrates for entity-specific semantic access	16
Gorno-tempini et al. 2001	Brain	Identification of faces and buildings: A functional neuroimaging study of semantically unique items	15
Evans et al. 2008	Depression and anxiety	A functional MRI study of amygdala responses to angry schematic faces in social anxiety disorder	11
Grabowski et al. 2001	Human brain mapping	A role for left temporal pole in the retrieval of words for unique entities	10
Grady et al. 1996	Human brain mapping	Effect of task difficulty on cerebral blood flow during perceptual matching of faces	10
Bolte et al. 2015	British journal of psychiatry	Training-induced plasticity of the social brain in autism spectrum disorder	25
Hooker et al. 2012	Schizophrenia research	Neural activity during emotion recognition after combined cognitive plus social cognitive training in schizophrenia	22
Scherf et al. 2007	Developmental science	Visual category-selectivity for faces, places and objects emerges along different developmental trajectories	30 (5-23 years)

Tseng et al. 2016	Human brain mapping	Differences in neural activity when processing emotional arousal and valence in autism spectrum disorders	84 (7–60 years)
Corradi-Dell'Acqua et al. 2014	Frontiers in human neurosciences	Neural responses to emotional expression information in high- and low-spatial frequency in autism: Evidence for a cortical dysfunction	15
Joseph et al. 2015	Journal of autism and developmental disorders	Typical and atypical neurodevelopment for face specialization: An fMRI study	23 (7 - 18 years)
Morita et al. 2016	Research in autism spectrum disorder	Neural correlates of emotion processing during observed self-face recognition in individuals with autism spectrum disorders	18
Young et al. 2015	Frontiers in human neuroscience	Synchrony between sensory and cognitive networks is associated with subclinical variation in autistic traits	47 (14-37 years)
Ishitobi et al. 2011	Research in autism spectrum disorder	Differential amygdala response to lower face in patients with autistic spectrum disorders: An fMRI study	24
Richey et al. 2015	Journal of autism and developmental disorders	Neural mechanisms of emotion regulation in Autism Spectrum Disorder	15
Chekho et al. 2012	PLoS ONE	Brain circuitries involved in semantic interference by demands of emotional and non-emotional distractors	24

CORRELATION BETWEEN REGIONAL ANTISACCADE RELATED ACTIVITY AND TASK PERFORMANCE

Supplementary Table 1.

Pearson correlations between individual Z max in the contrast Anti/Pro and percentage of errors or antisaccade reaction times. (In bold, are the p-values significant using FDR correction at p<0.05)

	ACC	LFEF	RFEF	LSPL	RSPL	RSMG
PERCENT ERRORS						
Pearson R	-0.279	-0.458	-0.444	-0.38	-0.442	-0.472
P value	**0.045**	**0.001**	**0.001**	**0.005**	**0.001**	**0.001**
Pearson R corr for age	-0.290	-0.487	-0.417	-0.361	-0.330	-0.287
P value	0.104	**0.004**	**0.014**	0.037	0.046	0.07
ANTISACC RT						
Pearson R	-0.293	-0.432	-0.457	-0.457	-0.539	-0.407
P value	**0.035**	**0.001**	**0.001**	**0.001**	**0.001**	**0.003**
Pearson R corr for age	-0.01	-0.392	-0.404	-0.418	-0.468	-0.297
P value	0.064	**0.005**	**0.003**	0.002	0.001	0.02

SPL = superior parietal lobule, FEF = frontal eye fields, SMG = supramarginal gyrus;

Supplementary Table 2. *Results from Bayesians linear regressions (1 per case) on the association between peak Z values and antisacades direction errors (percent errors) or latencies (saccadic reaction time, SRT), in each region of the antisaccade network. BF10 indicate the odds in favor of a linear relations compared to no relation (null model) in each regression.*

	**CARS**			**FACES**	
	**Pearson's r**	**BF_{10}**		**Pearson's r**	**BF_{10}**
**<u>PERCENT ERRORS</u>**					
ACC	-0.17	0.349		-0.261	0.948
lFEF	-0.278	1.2		-0.418	17.566
rFEF	-0.385	8.306		-0.428	22.551
lSPL	-0.325	2.527		0.307	
rSPL	-0.348	3.858		-0.434	26.016
rSMG	-0.252	0.839		-0.46	52.266

ANTISACC RT					
ACC	-0.301	1.391		-0.282	1.277
lFEF	-0.338	1.706		-0.379	7.177
rFEF	-0.435	3.221		-0.445	35.098
lSPL	-0.375	27.296		-0.362	5.122
rSPL	-0.453	6.616		-0.534	551.695
rSMG	-0.277	43.827		-0.38	7.383

SUPPLEMENTARY RESULTS FOR THE MVPA ANALYSIS

Supplementary Table 3

Task type decoding in ROI, in each age group. Percent correct classification averages are presented, as well as confidence intervals (CI). Classification was compared to chance level (50%) in each ROI, then corrected for multiple comparisons using FDR method. T values, degrees of freedom (df), uncorrected p value as well as corrected p value using FDR method. Significance levels are represented with a "" sign when significance (p<0.05) is reached after FDR correction.*

ROI	Group	Mean	CI	t	df	P	pFDR
**ACC**	Children	52.66	51.02 - 54.31	3.49	13	0.004	0.005*
	Adolescents	54.40	53.36 - 55.43	8.89	18	5.27e-08	1.73e-07*
	Adults	54.47	52.63 - 56.31	5.10	18	7.54e-05	1.29E-04*
**lSPL**	Children	58.77	55.42 - 62.12	5.65	13	7.87e-05	1.29E-04*
	Adolescents	60.07	57.78 - 62.36	9.23	18	3.02e-08	1.09E-07*
	Adults	63.78	60.84 - 66.72	9.85	18	1.13e-08	4.52E-08*
**rSPL**	Children	55.39	52.47 - 58.31	3.98	13	0.002	0.002*
	Adolescents	56.91	55.44 - 58.38	9.90	18	1.05e-08	4.52E-08*
	Adults	57.88	55.72 - 60.04	7.66	18	4.54e-07	1.26E-06*
**lFEF**	Children	54.62	52.45 - 56.80	4.60	13	0.0005	7.26E-04
	Adolescents	55.77	53.944 - 57.60	6.64	18	3.13e-06	6.27E-06*
	Adults	57.01	55.02 - 58.99	7.40	18	7.26e-07	1.87E-06*
**rFEF**	Children	57.49	54.36 - 60.63	5.17	13	0.0002	2.84E-04*
	Adolescents	58.95	56.53 - 61.37	7.77	18	3.68e-07	1.1E-06*
	Adults	62.63	59.99 - 65.27	10.04	18	8.45e-09	4.34E-08*
**rSMG**	Children	52.09	50.47 - 53.72	2.78	13	0.016	0.02*
	Adolescents	53.73	52.25 - 55.22	5.30	18	4.89e-05	8.80E-05*
	Adults	54.95	53.47 - 56.43	7.03	18	1.45e-06	3.08E-06*
**rFusiform**	Children	68.72	65.85 - 71.59	14.10	13	2.97e-09	1.78E-08*
	Adolescents	73.13	69.08 - 77.17	12.02	18	4.89e-10	3.52E-09*
	Adults	79.44	76.21 - 82.66	19.17	18	2.0e-13	3.60E-12*
**lFusiform**	Children	71.56	68.96 - 74.16	17.89	13	1.54e-10	1.39E-09*
	Adolescents	78.26	73.73 - 82.80	13.08	18	1.24e-10	1.39E-09*
	Adults	83.89	80.46 - 87.32	20.75	18	5.09e-14	1.83E-12*
**lAmygdala**	Children	51.16	49.05 - 53.26	1.19	13	0.26	0.26
	Adolescents	51.93	50.55 - 53.31	2.94	18	0.009	0.01*
	Adults	52.71	51.26 - 54.15	3.93	18	0.001	0.001*
**rAmygdala**	Children	50.76	49.76 - 51.77	1.64	13	0.13	0.13
	Adolescents	51.78	50.81 - 52.76	3.84	18	0.002	0.002*
	Adults	52.18	50.97 - 53.38	3.80	18	0.001	0.002*
**rPrecentral**	Children	52.67	50.75 - 54.60	3.00	13	0.01	0.01*
	Adolescents	53.75	52.47 - 55.02	6.18	18	7.87e-06	1.49E-05*
	Adults	56.03	54.28 - 57.78	7.25	18	9.66e-07	2.17E-06*
**rInsula**	Children	52.19	50.42 - 53.95	2.67	13	0.02	0.02

Supplementary Table 4. Stimulus type decoding in ROI, in each age group. Percent correct classification averages are presented, as well as confidence intervals (CI). Classification was compared to chance level (50%) in each ROI, then corrected for multiple comparisons using FDR method. T values, degrees of freedom (df), uncorrected p value as well as corrected p value using FDR method

ROI	Group	Mean	CI	t	df	p	FDR corrected
**ACC**	Children	49.30	47.83 - 50.76	1.03	13	0.32	0.48
	Adolescents	51.09	49.70 - 52.49	1.65	18	0.12	0.27
	Adults	49.00	47.68 – 50.33	1.58	18	0.13	0.27
**lSPL**	Children	48.51	46.48 – 50.53	1.59	13	0.14	0.27
	Adolescents	50.94	49.76 – 52.11	1.68	18	0.11	0.27
	Adults	49.51	48.10 – 50.90	0.75	18	0.46	0.64
**rSPL**	Children	50.96	49.62 – 52.29	1.55	13	0.14	0.27
	Adolescents	49.53	47.95 – 51.11	0.62	18	0.54	0.65
	Adults	50.52	48.79 – 52.23	0.63	18	0.54	0.65
**lFEF**	Children	48.07	46.38 – 49.76	2.47	13	0.03	0.15
	Adolescents	51.15	49.94 – 52.36	1.99	18	0.06	0.21
	Adults	49.23	47.64 – 50.82	1.02	18	0.32	0.48
**rFEF**	Children	48.87	47.53 – 50.22	1.80	13	0.09	0.26
	Adolescents	49.77	48.47 – 51.08	0.36	18	0.72	0.79
	Adults	51.01	49.91 – 52.10	1.93	18	0.07	0.21
**rSMG**	Children	48.69	47.29 – 50.09	2.015	13	0.07	0.21
	Adolescents	50.29	48.68 – 51.89	0.37	18	0.71	0.79
	Adults	49.99	48.94 – 51.05	0.01	18	0.99	0.99
**rFusiform**	Children	54.62	51.98 – 57.26	3.78	13	0.003	0.02*
	Adolescents	59.27	55.52 – 63.02	5.19	18	6.16e-05	0.0006*
	Adults	57.83	55.60 – 60.06	7.38	18	7.63e-07	1.00 e-05 *
**lFusiform**	Children	53.33	50.67 – 55.98	2.71	13	0.02	0.11
	Adolescents	59.36	55.88 – 62.83	5.66	18	2.28e-05	0.0003*

	Adults	59.18	56.89 – 61.47	8.43	18	1.16e-07	4.00 e-06 *
**lAmygdala**	Children	48.94	47.33 – 50.55	1.42	13	0.18	0.31
	Adolescents	49.84	48.50 – 51.18	0.25	18	0.80	0.84
	Adults	51.44	49.88 – 53.00	1.93	18	0.07	0.21
**rAmygdala**	Children	49.91	48.17 – 51.64	0.12	13	0.91	0.93
	Adolescents	48.97	47.51 – 50.44	1.48	18	0.16	0.28
	Adults	49.54	48.04 – 51.04	0.64	18	0.53	0.65
**rPrecentral**	Children	49.01	47.34 – 50.68	1.28	13	0.22	0.36
	Adolescents	49.52	48.05 – 50.99	0.68	18	0.50	0.65
	Adults	51.10	49.65 – 52.54	1.59	18	0.14	0.27
**rInsula**	Children	49.68	48.11 – 51.25	0.45	13	0.66	0.77
	Adolescents	50.48	49.14 – 51.83	0.76	18	0.46	0.64
	Adults	51.70	49.96 – 53.45	2.05	18	0.055	0.21

Significance levels are represented with a "*" sign when significance is reached after FDR correction

Supplementary Table 5. Stimulus type decoding in ROI, group differences. Percent correct classification was compared between age groups using ANOVA. FDR method was applied for multiple comparisons

ROI	F value	FDR corrected
**ACC**	$F(2, 49) = 3.121$, $p = 0.05$	0.13
**lSPL**	$F(2, 49) = 2.895$, $p = 0.06$	0.13
**rSPL**	$F(2, 49) = 0.9$, $p = 0.41$	0.45
**lFEF**	$F(2, 49) = 4.723$, $p = 0.01$	0.08
**rFEF**	$F(2, 49) = 3.142$, $p = 0.05$	0.13
**rSMG**	$F(2, 49) = 1.495$, $p = 0.23$	0.28
**rFusiform**	$F(2, 49) = 2.489$, $p = 0.09$	0.16
**lFusiform**	$F(2, 49) = 5.486$, $p = 0.007$	0.08
**lAmygdala**	$F(2, 49) = 3.069$, $p = 0.06$	0.13
**rAmygdala**	$F(2, 49) = 0.395$, $p = 0.68$	0.68

**rPrecentral**	F(2, 49) = 2.284, p = 0.11	0.17
**rInsula**	F(2, 49) = 1.801, p = 0.18	0.23
